# XVII International AIDS Conference: From Evidence to Action - AIDS 2008 and the global response to AIDS

**DOI:** 10.1186/1758-2652-12-S1-S7

**Published:** 2009-10-06

**Authors:** Rodney Kort

**Affiliations:** 1 Kort Consulting, Toronto, Canada

## Abstract

The impact of the XVII International AIDS Conference (AIDS 2008) was reflected in a number of commitments from political and business leaders, who announced initiatives ranging from implementing comprehensive sexual education for young people in Latin America to reducing regulatory barriers and the price of drugs in the host country. The unprecedented media coverage brought attention and public awareness to the epidemic in Latin America.

Several meetings and sessions at AIDS 2008 also addressed the potential for the International AIDS Conference to play an even stronger role in tracking progress towards universal access and in improving accountability in the global response to AIDS, particularly given some of the inherent weaknesses in the United Nations General Assembly Special Session (UNGASS) review process. The impact of AIDS 2008 was strongest in Mexico, the host country, and in Latin America. Highlights included the policy changes announced by President Calderon on pharmaceutical manufacturing to the focus on sex workers and gay and other MSM in marches, activism and the conference programme.

The next two years will determine whether the successes reported in Mexico are sustained and whether there is progress in addressing the barriers that continue to hamper an evidence-based response to HIV/AIDS. The next International AIDS Conference is scheduled for the universal address deadline of 2010.

## Discussion

### Tracking progress and strengthening accountability

In an effort to strengthen the role of the conference as an accountability mechanism, the AIDS 2008 Conference Coordinating Committee organized several sessions and meetings that focused on tracking progress on existing commitments and developing strategies for increased accountability among stakeholders. In a session reviewing progress on UNGASS targets, several speakers reminded participants that, while the UNGASS process has been essential to scale-up efforts, it is hampered by the fact that both the goals themselves and the reports on progress towards meeting the commitments are drafted by UN Member States, over which both multilateral institutions and civil society have limited influence [1]. Kieran Daly observed that many countries do not report on UNGASS indicators related to policies and laws prohibiting discrimination and protecting key populations, and that data on some most at risk populations are often absent from such reports [2]. While 147 Member States submitted reports for the most recent UNGASS meeting in 2008, only one submitted data on all 25 indicators and more than 40 countries did not submit any national progress reports [3,4]. The intransigence among some Member States to specifically identify the populations most vulnerable to HIV in the 2001 and 2006 UNGASS declarations â€“ or report on them in subsequent progress reports â€“ is only one example of the limitations of the UNGASS process. The absence of such information compromises efforts to address some of the underlying drivers of the epidemic.

As a multidisciplinary meeting that brings together scientific, political and community leadership as equal partners in the global response, the conference has inherent advantages compared to UNGASS meetings, in which government officials from Member States ultimately determine the commitments and key messages. The current size and complexity of the conference programme, however, brings with it significant challenges to restructuring part or all of the conference to serve as a more formal accountability mechanism, with a more systematic monitoring component. If â€“ as some speakers suggested â€“ the conference is evolving into a broader health and development meeting, it will doubtless add additional complexity to such an effort.

At a meeting of key stakeholders at the conference hosted by the IAS, there was strong support for a more strategic and structured approach to monitoring progress on universal access targets and Millennium Development Goals (MDGs), particularly at AIDS 2010, which coincides with the deadline for meeting UNGASS commitments. However, the suggestion that the conference issue report cards on a select group of countries using a set of core indicators, an approach similar to that proposed by AIDS Accountability International [5], raised concerns about a parallel UNGASS process when the UN system has already invested heavily in infrastructure and capacity building to support country-level reporting on the UNGASS National Composite Policy Index.

Potential strategies for formalizing the role of the International AIDS Conference as an accountability mechanism include establishing a separate accountability track or restructuring the Leadership Programme to serve this purpose. The issue of securing the participation of senior political leaders in a forum where their commitments to most at risk populations, controversial prevention interventions (such as drug consumption rooms), or structural drivers of the epidemic will be scritinized, is an ongoing challenge for conference organizers [6]. IAS Executive Director Craig McClure agreed that the existing charter, which outlines the roles and responsibilities of the CCC and other governance structures of the International AIDS Conference, may need to be reviewed. While there is substantial interest in shaping AIDS 2010 into a more formal accountability mechanism, the likelihood of drawing senior government officials into a forum with a strong activist presence and many opportunities for public criticism will remain a challenge for the IAS and its co-organizers.

**Figure 1:**
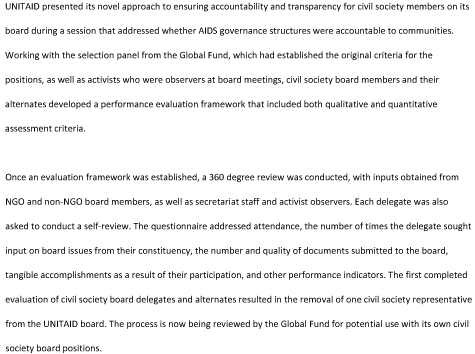
Civil Society Accountability: UNI TAID

Not all accountability discussions focused on political leaders and government policymakers; one session included an innovative performance evaluation process established by civil society representatives on the UNITAID board to assess civil society accountability (Figure 1) [7,8].

### Strengthening links between conferences

**Figure 2:**
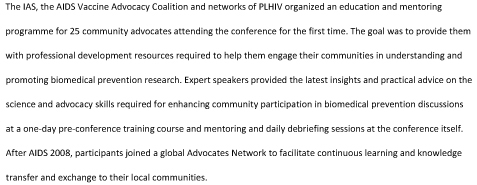
Education and Mentoring Programme

The plethora of regional AIDS conferences and other scientific meetings is presenting donors and HIV professionals with increasingly difficult decisions about whether to fund and participate in these resource-intensive events. The IAS has devoted significant work and resources to regional partnerships over the last several years and is providing financial, organizational and technical support â€“ with support from the Bill & Melinda Gates Foundation â€“ to several regional conferences. The purpose of this approach is not only to strengthen knowledge transfer between these conferences and the larger International AIDS Conference â€“ through such mechanisms as the regional sessions organized for AIDS 2008 â€“ but to leverage donor investments in knowledge exchange and networking while ensuring that regional-specific issues are adequately addressed. The IAS has also expanded its own professional development activities, partnering with other agencies to strengthen learning opportunities (Figure 2).

At a meeting that focused on how to strengthen the impact of international and regional AIDS conferences in the global response, several speakers noted the growing scepticism among media and experienced delegates about the benefits of these meetings relative to their cost, and emphasized the need to more clearly differentiate the respective merits and comparative advantage of each meeting [9]. This was underscored by a post-conference report on media coverage indicating that some major media outlets were relying on local news bureaus and wire services for coverage rather than sending specialist reporters to cover the conference, at least partly due to cost considerations [10]. Relatively new conferences, such as the PEPFAR-initiated HIV/AIDS Implementers Meeting, are adding to the complex topography of HIV-related meetings and conferences. While interest and participation in the International AIDS Conference has remained high, several observers suggested that the timetable of regional and international AIDS conferences â€“ as well as other scientific meetings â€“ be reviewed and amalgamated where feasible [11]. The challenges of such a process, in a context where each conference includes multiple organizing partners and competing agendas and financial interests, is formidable. Challenges notwithstanding, the IAS indicated its support for such an initiative and UNAIDS agreed to use its convening power to facilitate follow-up discussions in an effort to move the field towards a more rational and cost-effective approach to these meetings.

### The media and the message

One of the primary goals of the International AIDS Conference is to raise HIV awareness among the general public, policymakers and other decision-makers through widespread media coverage. Almost 2,500 journalists filed stories on every aspect of the conference and affiliated activities, with over 11,000 stories filed in English language print media alone.

One of the most important functions of the conference is to increase awareness of HIV in communities around the world, through stories that address every aspect of the response to AIDS. Although it is difficult to assess the impact of this coverage on the global response to AIDS, the prospect of negative media coverage almost certainly ensures attention from political leaders for whom the media is an essential conduit of public opinion. With this in mind, several policy or regulatory changes were announced by both political and business leaders immediately prior to or during AIDS 2008, including:

• The President of Panama repealed the law which made sex between men a criminal act, the last country in Latin America (not including the Caribbean) to remove homosexual acts from the criminal code.

• Mexican President Felipe Calderon announced removal of the regulatory barrier which required pharmaceutical companies to have a manufacturing plant in Mexico in order to sell their drugs in the country, and to making low drug pricing a priority for his government.

• Mexico City Mayor Marcello Ebrard spoke out against homophobia in his Closing Session speech and announced that local government will be distributing new text books on health that address sex education in public schools.

• Indian Health Minister Anbumani Ramadoss publicly urged the Indian parliament to repeal section 377 of its criminal codes, which criminalizes 'unnatural acts' (including homosexuality), reinforcing an earlier statement to that effect by India's High Court Judge, Bilal Nazki.

• China announced that it would lift its ban prohibiting people living with HIV from entering the country.

• The Coalition of First Ladies and Women Leaders of Latin America on HIV announced their commitment to eliminating MTCT and syphilis by 2015.

• Representatives from 30 Ministries of Health and 25 Ministries of Education in Latin America announced they would prioritize HIV prevention education and sex education in schools as part of their regional strategy on HIV/AIDS.

• Merck and Company announced it would cut its price of Stocrin (efavirenz) in Mexico by 40% from 777 pesos per patient monthly to 468 pesos (roughly from US $77.50 to US $46), and on Isentress (raltegravir) by 30% from 9.05 pesos to 6.85 pesos per patient monthly (approximately US $903 to US $683).

• Spanish Vice President Maria Teresa Fernandez de la Vega announced her country's contribution of €10.2 million to UNAIDS, of which €3 million will support the activities of the International AIDS Vaccine Initiative and €1.5 million will go to the International Partnership for Microbicides.

• The Spanish government announced the inclusion of lipoatrophy treatment to the list of services covered by the National Health System.

Beyond the tangible policy and funding announcements, analysis of the news coverage revealed an unprecedented amount of media attention to stigma and discrimination, particularly related to the Opening Session keynote on this issue by UN Secretary-General Ban-Ki moon. Other topics that received considerable media attention internationally include the challenges faced globally by gay and other MSM, and the ongoing debate regarding whether AIDS receives a disproportionate amount of funding relative to other health and development issues. Coverage by Mexican and other Latin American media focused particularly on barriers to delivering prevention, care and treatment to gay and other MSM, an issue which emerged as a dominant theme of AIDS 2008.

The need to address homophobia and the challenges faced by the gay and other MSM populations was underlined in a statement released by the Episcopal Social Pastoral Commission of the Mexican Catholic Church. The statement condemned the stigma and discrimination faced by PLHIV and pledged to work with other social actors in the response, particularly those targeting socially vulnerable communities, 'such as indigenous people, women, prisoners, young people, those excluded from the education and health systems, rural dwellers, migrants and their families, children and young homeless people.'[12] Notably absent from the list was the group with the highest HIV prevalence of any population in Mexico: gay and other MSM.

South African news coverage focused on conference sessions that addressed the dangers of criminalizing HIV, covering Justice Edwin Cameron's plenary speech on this topic, as well as the promising potential of task-shifting to address health system capacity issues in low and middle-income countries.

**Figure 3:**
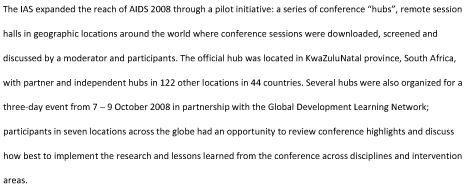
Conference Hubs

As part of its ongoing strategy to expand the reach and impact of the conference through use of the Internet, the IAS relied on its online partners Clinical Care Options, which once again provided official online scientific coverage, and Kaisernetwork.org, which produced over 75 webcasts, daily news reports, and other news summaries to a global online audience. It also initiated a pilot project aimed at expanding the reach of the conference to those unable to attend via conference hubs (Figure 3).

## Conclusion

AIDS 2008 addressed some of the most pressing issues facing the HIV field as it moves ever closer to the 2010 deadline for meeting universal access targets. Among the many tangible impacts of the conference, some of the most important are: recognition of the need for policymakers to take steps to move beyond rhetorical commitments to human rights for vulnerable populations to concrete legislative and policy changes that will have an impact on the environmental and structural drivers of the epidemic; new evidence that the health system-strengthening effects of HIV-specific funding can lead to greater synergies with other areas of the health care system and help deconstruct the polemic of vertical versus horizontal investments; a new consensus on the need for 'combination prevention', which integrates existing biomedical, behavioural and community based approaches with interventions that address the social and structural inequalities which continue to drive infections; new clinical evidence suggesting that ART should be initiated earlier and used aggressively to curb mortality and morbidity, and to maximize its potential impact on reducing transmission; the promise of PrEP as a potential new biomedical prevention tool on the horizon; and new basic science research that is exploring cellular dynamics in the inflammatory response to HIV that could lead to new therapeutic applications and - perhaps- new insights into viral eradication.

The next two years will determine whether the advances reported at AIDS 2008 are sustained and how much progress will be made on the necessary policy and legislative changes to achieve universal access by 2010, the next year of the International AIDS Conference.

## Competing interests

Rodney Kort is an independent consultant contracted by the International AIDS Society for the purpose of preparing and editing the AIDS 2008 Impact Report for publication.

## Authors' contributions

Rodney Kort drafted this text and has approved the manuscript for publication.
